# Heterozygous deletion of *Cul4b* in female mice leads to ovulatory dysfunction and female infertility

**DOI:** 10.1016/j.gendis.2024.101381

**Published:** 2024-07-24

**Authors:** Yufeng Wang, Yuting Liu, Wei Jiang, Yu Song, Yongxin Zou, Molin Wang, Qiao Liu, Gongping Sun, Yaoqin Gong, Baichun Jiang

**Affiliations:** a The Key Laboratory of Experimental Teratology of the Ministry of Education and Department of Genetics, School of Basic Medical Sciences, Cheeloo College of Medicine, Shandong University, Jinan, Shandong 250012, China; b The Key Laboratory of Experimental Teratology of the Ministry of Education and Department of Histology and Embryology, School of Basic Medical Sciences, Cheeloo College of Medicine, Shandong University, Jinan, Shandong 250012, China

It is estimated that infertility impacts 8%–12% of reproductive-aged couples worldwide. Female infertility accounts for 37% of causes among infertile couples, and ovulatory dysfunction is regarded as its most common factor.^1^ CUL4B belongs to the Cullin family, whose members are the scaffolding proteins of Cullin-RING E3 ligases (CRLs). Human *CUL4B* gene mutations result in X-linked mental retardation syndromes. In addition to mental retardation, patients have symptoms such as short stature, obesity, and hypogonadism. Global (*Sox2-Cre*) or germ cell-specific (*Vasa-Cre*) *Cul4b* knockout male mice are infertile with impaired spermatozoa motility and spermatogonial stemness. DDB1 and DCAF1, two members of the CRL4A/B complex, can regulate oocyte survival, reprogramming,^2^ and meiotic maturation of oocytes.^3^ In this study, we generated *Sox2-Cre*^*+/−*^;*Cul4b*^*f/+*^ heterozygous female mice and found that these mice were infertile due to anovulation. CUL4B affects granulosa cell number and follicle development by regulating the follicle-stimulating hormone (FSH)/aromatase/estrogen loop. These results reveal a new function of CUL4B in follicular development and ovulation and provide a novel theoretical basis for the diagnosis and treatment of ovulation dysfunction and female infertility.

In this study, to investigate CUL4B function in female reproduction, we purposed to generate female *Cul4b* knockout mice using *Sox2*-*Cre*. Surprisingly, the *Sox2-Cre*^*+/−*^;*Cul4b*^*f/+*^ heterozygous female mice were infertile (Fig. 1A). There was no fetus in the uterus of *Sox2-Cre*^*+/−*^;*Cul4b*^*f/+*^ mice at 7.5 days post coitum (Fig. S1). Hematoxylin and eosin staining showed that corpora lutea were not observed in *Sox2-Cre*^*+/−*^;*Cul4b*^*f/+*^ mouse ovaries. In striking contrast, the ovaries of *Sox2-Cre*^*+/−*^;*Cul4b*^*f/+*^ mice harbored numerous unruptured follicles with trapped oocytes (Fig. 1B). These data indicated that there was no ovulation in *Sox2-Cre*^*+/−*^;*Cul4b*^*f/+*^ mice. While *Cul4b*^*f/+*^ control mice had regular estrous cycles averaging 4–5 days, the estrus cycles of *Sox2-Cre*^*+/−*^;*Cul4b*^*f/+*^ mice were irregular, with prolonged periods of metestrus and diestrus (Fig. 1C, D). The serum levels of FSH, luteinizing hormone, estradiol, and progesterone were decreased in *Sox2-Cre*^*+/−*^;*Cul4b*^*f/+*^ mice, while the serum level of testosterone was increased (Fig. S2). These results suggested that *Sox2-Cre*^*+/−*^;*Cul4b*^*f/+*^ heterozygous female mice were infertile due to anovulation.

**Figure 1:**
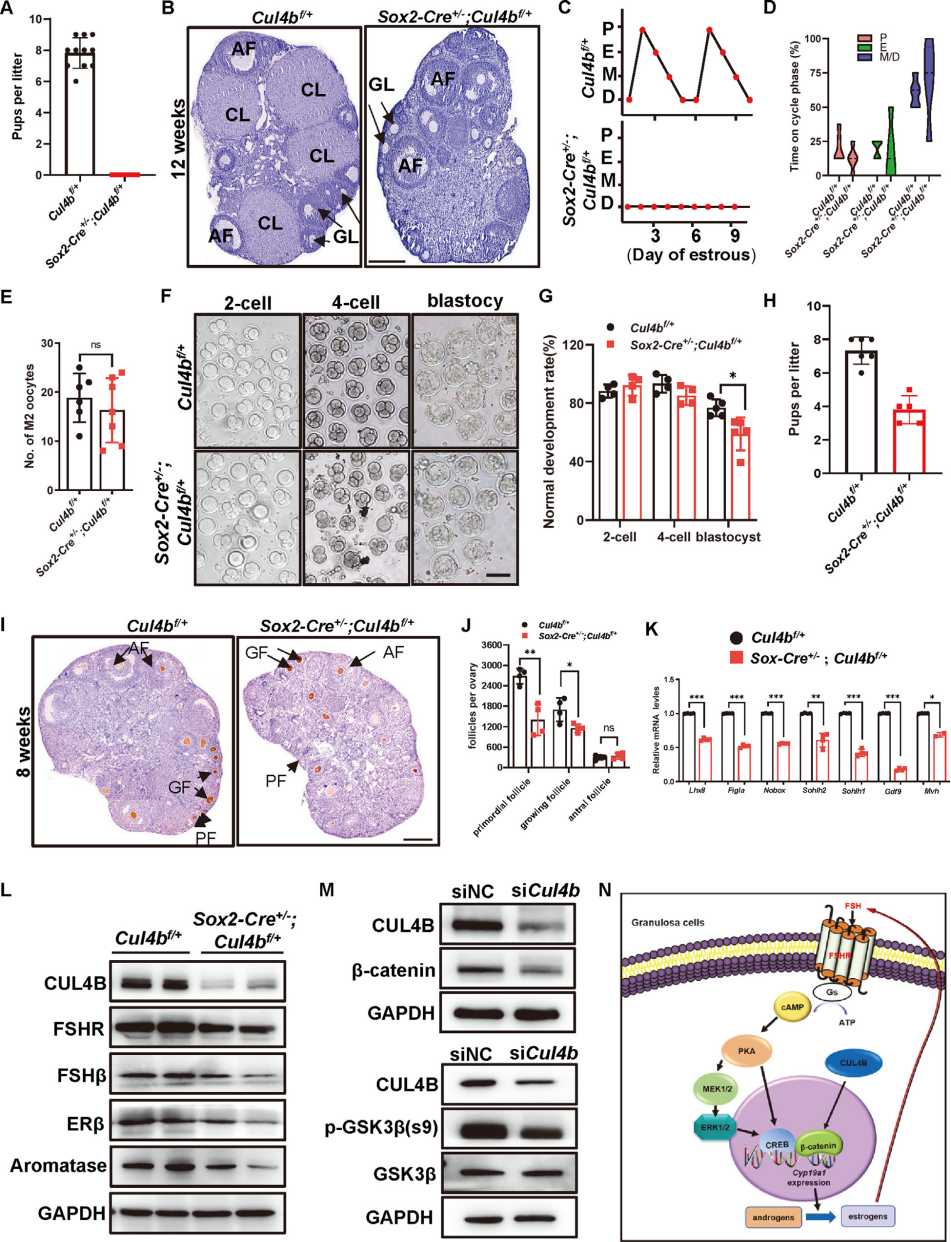
Heterozygous deletion of *Cul4b* in female mice leads to ovulatory dysfunction and female infertility via follicle-stimulating hormone (FSH)/aromatase/estrogens loop. **(A)** Litter size of *Cul4b*^*f/+*^ and *Sox2-Cre*^*+/−*^;*Cul4b*^*f/+*^ mice in a breeding trial for 6 months. **(B)** Representative hematoxylin and eosin staining of ovaries from 12-week-old *Cul4b*^*f/+*^ and *Sox2-Cre*^*+/−*^;*Cul4b*^*f/+*^ mice. AF, antral follicles; CL, corpus luteum; GF, growing follicles. Scale bar, 100 μm. **(C)** Typical estrous cycles are shown for 8-week-old *Cul4b*^*f/+*^ and *Sox2-Cre*^*+/−*^;*Cul4b*^*f/+*^ mice. **(D)** The corresponding estrous cycle violin diagram (*n* = 6). D, diestrus; M, metestrus; E, estrus; P, proestrus. **(E)** The number of metaphase II (MII) oocytes retrieved from oviducts of *Cul4b*^*f/+*^ and *Sox2-Cre*^*+/−*^;*Cul4b*^*f/+*^ mice after superovulation. ns, no significance. **(F)** Morphology of 2-cell, 4-cell, and blastula embryos from *in vitro* fertilized oocytes of *Cul4b*^*f/+*^ and *Sox2-Cre*^*+/−*^;*Cul4b*^*f/+*^ mice. Scale bar, 100 μm. **(G)** Percentages of normal 2-cell, 4-cell, and blastula embryos. **(H)** Litter size of *Cul4b*^*f/+*^ and *Sox2-Cre*^*+/−*^;*Cul4b*^*f/+*^ mice after superovulation. **(I)** Representative immunohistochemistry images of MVH in the ovaries of *Cul4b*^*f/+*^ and *Sox2-Cre*^*+/−*^;*Cul4b*^*f/+*^ mice at 8 weeks. AF, antral follicles; GF, growing follicles; PF, primordial follicles. Scale bar, 100 μm. **(J)** The numbers of primordial, growing, and antral follicles per ovary in *Cul4b*^*f/+*^ and *Sox2-Cre*^*+/−*^;*Cul4b*^*f/+*^ mice are shown. **(K)** Relative mRNA levels of oocyte-specific genes *Lhx8*, *Figlα*, *Nobox*, *Sohlh1*, *Sohlh2*, *Gdf9*, and *Mvh* in the ovaries of *Cul4b*^*f/+*^ and *Sox2-Cre*^*+/−*^;*Cul4b*^*f/+*^ mice were determined by quantitative PCR. **(L)** FSHR, FSHbeta, ERbeta, and aromatase protein levels in the ovaries of 8-week-old *Cul4b*^*f/+*^ and *Sox2-Cre*^*+/−*^;*Cul4b*^*f/+*^ mice were determined by Western blotting. **(M)** beta-catenin protein levels and phosphorylation of GSK3beta at Ser9 in the control and *Cul4b*-knockdown KGN cells were determined by Western blotting. **(N)** A model by which CUL4B regulates the FSH/aromatase/estrogens loop. Lack of CUL4B leads to impaired FSH/cAMP pathway and decreased expression of *Cyp19a1* in granulosa cells, which cause increased androgens and decreased estrogens. Estrogens synthesized in granulosa cells in turn regulate gonadotropin secretion in the pituitary.

Then, we studied the ability of ovulation in response to gonadotropin treatment in *Sox2-Cre*^*+/−*^;*Cul4b*^*f/+*^ mice. After superovulation with PMSG (pregnant mare's serum gonadotrophin) and hCG (human chorionic gonadotrophin), α-Tubulin staining showed that the spindles of oocytes were normally separated in both *Sox2-Cre*^*+/−*^;*Cul4b*^*f/+*^ and *Cul4b*^*f/+*^ control mice (Fig. S3). Similar numbers of metaphase II oocytes were retrieved from the oviducts of *Sox2-Cre*^*+/−*^;*Cul4b*^*f/+*^ and *Cul4b*^*f/+*^ control mice (Fig. 1E). *In vitro* fertilization experiments showed that most metaphase II oocytes from both *Sox2-Cre*^*+/−*^;*Cul4b*^*f/+*^ and *Cul4b*^*f/+*^ control mice could be fertilized and developed to the blastocyst phase, although the number of blastocysts from *Sox2-Cre*^*+/−*^;*Cul4b*^*f/+*^ mice was less than that from *Cul4b*^*f/+*^ control mice (Fig. 1F, G). Importantly, when the superovulated *Sox2-Cre*^*+/−*^;*Cul4b*^*f/+*^ and *Cul4b*^*f/+*^ control mice were mated with male mice, the *Sox2-Cre*^*+/−*^;*Cul4b*^*f/+*^ mice gave birth to live pups, although the number of pups was less than that of the *Cul4b*^*f/+*^ control mice (Fig. 1H). These results suggested that the ovaries of *Sox2-Cre*^*+/−*^;*Cul4b*^*f/+*^ mice could respond to FSH and luteinizing hormone treatment and had the ability to ovulate.

Furthermore, both the mRNA and protein levels of CUL4B were reduced in the ovaries of *Sox2-Cre*^*+/−*^;*Cul4b*^*f/+*^ mice compared with those in *Cul4b*^*f/+*^ mice (Fig. S4A, B). Surprisingly, immunohistochemical staining showed that the expression of CUL4B was markedly reduced in the granulosa cells of *Sox2-Cre*^*+/−*^;*Cul4b*^*f/+*^ mice but was not changed in the oocytes (Fig. S4C), which was possibly due to skewed X-chromosome inactivation in the granulosa cells of the mice. Continuous section and FOXO1 (granulosa cell marker) staining of the ovaries showed that the number of granulosa cells in the follicles of *Sox2-Cre*^*+/−*^;*Cul4b*^*f/+*^ mice was greatly reduced (Fig. S4D, E). The protein level of FOXO1 was also decreased in the ovaries of *Sox2-Cre*^*+/−*^;*Cul4b*^*f/+*^ mice (Fig. S4F). The EdU incorporation assay showed that the percentage of EdU-positive cells per follicle in *Sox2-Cre*^*+/−*^;*Cul4b*^*f/+*^ mice was significantly decreased (Fig. S5A). Consistent with the *in vivo* results, the proliferation of primary granulosa cells was also reduced after knocking down *Cul4b* expression (Fig. S5B). On the other hand, the TUNEL assay showed that the number of apoptotic cells was greatly increased in the ovaries of *Sox2-Cre*^*+/−*^;*Cul4b*^*f/+*^ mice (Fig. S5C). The proapoptotic ratio of Bax/Bcl-2 was significantly increased in the ovaries of *Sox2-Cre*^*+/−*^;*Cul4b*^*f/+*^ mice (Fig. S5D–F). These results suggested that the ovulatory dysfunction and infertility of *Sox2-Cre*^*+/−*^;*Cul4b*^*f/+*^ mice was due to decreased expression of CUL4B in granulosa cells, leading to reduced granulosa cells in the follicles.

In addition, we evaluated whether the reduced number of granulosa cells has an impact on ovarian follicle development. Continuous section and MVH (oocyte marker) staining of ovaries revealed that the numbers of both primordial follicles and growing follicles were reduced in the ovaries of *Sox2-Cre*^*+/−*^;*Cul4b*^*f/+*^ mice compared with those in *Cul4b*^*f/+*^ mice, while the number of antral follicles was not changed (Fig. 1I, J; Fig. S6A), indicating that folliculogenesis was retarded. The mRNA and protein levels of the oocyte-specific genes *Lhx8*, *Figlα*, *Nobox*, *Sohlh1*, *Sohlh2*, *Gdf9*, and *Mvh* were significantly decreased in the ovaries of *Sox2-Cre*^*+/−*^;*Cul4b*^*f/+*^ mice (Fig. 1K; Fig. S6B). These results suggested that follicle development was impaired in the ovaries of *Sox2-Cre*^*+/−*^;*Cul4b*^*f/+*^ mice.

Next, to further examine the molecular mechanism underlying the infertility of *Sox2-Cre*^*+/−*^;*Cul4b*^*f/+*^ mice, we determined whether *Cul4b* deletion affected steroidogenesis in mouse ovaries. The mRNA and protein levels of *Fshr*, *Erβ*, *Star*, and *Cyp19a1* (encodes aromatase) were significantly reduced in the ovaries of *Sox2-Cre*^*+/−*^;*Cul4b*^*f/+*^ mice compared with those in *Cul4b*^*f/+*^ mice (Fig. 1L; Fig. S7A). We then examined the phosphorylation of intracellular effectors of the FSH/cAMP pathway, and the results showed that the levels of p-PKA, p-CREB, p-MEK, and p-ERK were all reduced in the ovaries of *Sox2-Cre*^*+/−*^;*Cul4b*^*f/+*^ mice compared with those in *Cul4b*^*f/+*^ mice (Fig. S7B). A previous study showed that beta-catenin was indispensable for FSH/cAMP-mediated regulation of aromatase expression,^4^ and we previously found that CUL4B positively regulated beta-catenin via the AKT-GSKbeta axis.^5^ Therefore, we next examined the regulation of beta-catenin by CUL4B in KGN cells. Western blotting showed that the protein level of beta-catenin was decreased in *Cul4b* knockdown KGN cells (Fig. 1M). The level of p-GSK3beta (Ser9), an inactive form of GSK3beta, was significantly reduced in *Cul4b* knockdown KGN cells (Fig. 1M), indicating that the kinase activity of GSK3beta was increased, which phosphorylates beta-catenin for its degradation. Indeed, the reduction in beta-catenin in *Cul4b* knockdown KGN cells was greatly attenuated by the proteasome inhibitor MG132 (Fig. S7C). Moreover, SB216763, an inhibitor of GSK3beta, efficiently blocked the reduction in beta-catenin levels in *Cul4b* knockdown KGN cells (Fig. S7D). These results imply that beta-catenin may mediate the regulation of *Cyp19a1* expression by CUL4B. Finally, the protein levels of FSHR, FSHβ, ERβ, aromatase, p-PKA, p-CREB, p-MEK, and p-ERK were restored in the ovaries of superovulated *Sox2-Cre*^*+/−*^;*Cul4b*^*f/+*^ mice (Fig. S8A, B). These results suggested that CUL4B could regulate the FSH/aromatase/estrogen loop (Fig. 1N).

## Ethics declaration

All animal care and experiments were approved by the Animal Care and Use Committee of the School of Basic Medical Sciences of Shandong University (No. ECSBMSSDU2019-2-004).

## Author contributions

B.J. and Y.G. conceived the study concept and design; Y.W. performed most experiments; Y.L. and W.J. helped to set up the experiments; G.S., Y.Z., M.W., Y.S., and Q.L. provided acquisition, analysis, and interpretation of the data; B.J. wrote and revised the manuscript. All the authors read and approved the final manuscript.

## Funding

This work was supported by the 10.13039/501100001809National Natural Science Foundation of China (No. 31970559, 82171851, 32370652).

## Conflict of interests

The authors have no competing interests to declare.
