# Supplementary material for: Heterozygous deletion of Cul4b in female mice leads to ovulatory dysfunction and female infertility
Source: Genes Dis. 2024 Jul 24;12(1):101381. doi: 10.1016/j.gendis.2024.101381 (PMC11683949; doi:10.1016/j.gendis.2024.101381)
Supplement: Multimedia component 1 [file mmc1.docx]

**Supplementary Materials**

**Heterozygous deletion of *Cul4b* in female mice leads to ovulatory dysfunction and female infertility**

Yufeng Wang^a^, Yuting Liu^a^, Wei Jiang^a^, Yu Song^a^, Yongxin Zou^a^, Molin Wang^a^, Qiao Liu^a^, Gongping Sun^b^, Yaoqin Gong^a,**^, Baichun Jiang^a,*^

^a^ The Key Laboratory of Experimental Teratology of the Ministry of Education and Department of Genetics, School of Basic Medical Sciences, Cheeloo College of Medicine, Shandong University, Jinan, Shandong 250012, China.

^b^ The Key Laboratory of Experimental Teratology of the Ministry of Education and Department of Histology and Embryology, School of Basic Medical Sciences, Cheeloo College of Medicine, Shandong University, Jinan, Shandong 250012, China.

**E-mail addresses**: [yxg8@sdu.edu.cn](mailto:yxg8@sdu.edu.cn) (Y. Gong), [jiangbaichun@sdu.edu.cn](mailto:jiangbaichun@sdu.edu.cn) (B. Jiang)

**Supplementary Materials and Methods**

**Mice**

*Cul4b* floxed mice were generated as previously reported. Female *Cul4b^f/f^* mice were mated with male *Sox2-Cre* mice to generate *Sox2-Cre^+/-^*;*Cul4b^f/+^* female mice. The littermate *Cul4b^f/+^* female mice were used as controls. All mice were on a C57BL/6J background.

**Fertility test**

Sexually mature (6- to 8-week-old) female mice were mated with fertile male mice continuously for at least 6 months. The numbers of litters and pups were recorded.

**Assessment of estrous cycles**

For estrous cycle analysis, mice were individually housed with enrichment. Vaginal cytology was assessed for 30 consecutive days. Vaginal smears were fixed for H&E staining and microscopic examination.

**Ovarian histology and immunostaining**

The ovaries were fixed in 4% (W/V) paraformaldehyde in phosphate buffered saline (PBS) for 12 hours at 4 °C, dehydrated, embedded in paraffin and sectioned for H&E staining and immunostaining. Immunohistochemistry (IHC) and immunofluorescence (IF) were performed as previously described. Primary antibodies included anti-CUL4B (Sigma, 1:200), anti-α-tubulin (Abcam; 1:200), anti-MVH (Abcam, 1:200), and anti-FOXO1 (CST; 1:200). Secondary antibodies included goat anti-rabbit or mouse horseradish peroxidase (HRP), goat anti-rabbit or mouse FITC or Cy3 (Jackson ImmunoResearch, 1:200).

**Hormone analysis**

During diestrus, mice were anesthetized by intraperitoneal injection of avertin. Blood was retrieved by eyeball removal. Serum was separated by centrifuging at 3,000 rpm for 15 minutes and stored at -80 °C. Hormones were quantified using commercial Animalunion Biotechnology Kits.

**Superovulation experiments**

For superovulation, female mice were injected intraperitoneally with 5 IU of pregnant mare serum gonadotropin (PMSG) followed by 5 IU of human chorionic gonadotropin (hCG) 46 hours later. After an additional 13 hours, the mice were anesthetized, and then the ovaries and oviducts were collected. Oocytes were surgically removed from oviducts and digested with 0.1% hyaluronidase for removal of cumulus cells. The numbers of oocytes were counted.

***In vitro* fertilization (IVF)**

Oviducts were collected from the superovulated mice, and the cumulus oocyte complex (COC) was released and placed in fertilization droplets of human tubal fluid (HTF) medium. Sperm from healthy males were capacitated in HTF medium for 60 minutes and were added to the COCs and incubated in HTF medium for 8 hours at 37 °C under 5% CO_2_. Unbound sperm were washed away after incubation. Embryos were cultured in potassium simplex optimized medium (KSOM) (Millipore) 24 hours after fertilization.

**Quantitative real-time RT‒PCR (qPCR)**

Total RNA was extracted using TRIzol reagent (Invitrogen). Freshly isolated RNA was reverse transcribed to generate cDNA. The resulting cDNA was then subjected to quantitative real-time RT‒PCR using 2×ChamQ SYBR Color qPCR Master Mix (Vazyme) using a Light Cycler real-time PCR instrument (Roche, LC480, Basel, Switzerland). Three samples for each gene were performed, and each sample was repeated in triplicate. The quantified individual RNA expression levels were normalized to *Gapdh*. The sequences of the primers are listed in Supplementary Table 1.

**Western blotting**

Western blotting was performed as previously described. The primary antibodies are listed in Supplementary Table 2. Secondary antibodies included anti-rabbit or mouse horseradish peroxidase (HRP) (Abmart). Estimates of protein amounts were obtained by measuring the area of protein bands using ImageJ, and were normalized to the GAPDH protein amount in the respective samples.

**Primary granulosa cell culture**

The ovaries were collected from 3-week-old mice and washed three times with DMEM/F12 media (HyClone, SH30023.01B). The follicles were punctured with a 1 mL syringe to allow the granulosa cells to flow out naturally. The granulosa cells were cultured in media containing DMEM/F12, 10% fetal bovine serum (Gibco, 10099-141), 100 IU/mL penicillin G, and 100 mg/mL streptomycin sulfate.

**RNA interference**

Primary granulosa cells were transfected with siRNA using Lipo3000™ reagent (Thermo Fisher, L3000015). si-NC and si-*Cul4b* were customized at Gemma Company. The sequence of the siRNA oligonucleotide was 5'-CAAUCUCCUUGUUUCAGAATTUUCUGAAACAAGGAGAUUGTG-3'.

**EdU incorporation assay**

The percentage of ovarian somatic cells in S-phase was evaluated by EdU incorporation assay using the Cell-Light EdU DNA cell proliferation kit (Beyotime, C10371-1, Nanjing, China). EdU was injected intraperitoneally into mice at 20 mg per kg body weight. Mice were sacrificed after 15 minutes, and the ovaries were dissected and fixed in 4% paraformaldehyde. After staining, the slides were counterstained with Hoechst 33342 and visualized under a fluorescence microscope.

**TUNEL assay**

Apoptotic cell analysis was performed using a terminal deoxynucleotidyl transferase-mediated dUTP nick-end labeling (TUNEL) staining kit (Vazyme, Nanjing, China) following the manufacturer’s recommendations. After labeling, the slides were counterstained with DAPI and visualized under a fluorescence microscope.

**Statistical analysis**

All experiments were repeated at least three times, and the data are expressed as the mean ± SEM. Statistical tests and plots were performed using Prism 8 (GraphPad Software Inc.). Two sample t test was used to analyze data from two groups for any significant differences. A *p* value of less than 0.05 was considered statistically significant. * *p*<0.05, ** *p*<0.01; *** *p*<0.001.

**Supplementary Figures and** **Figure legends**


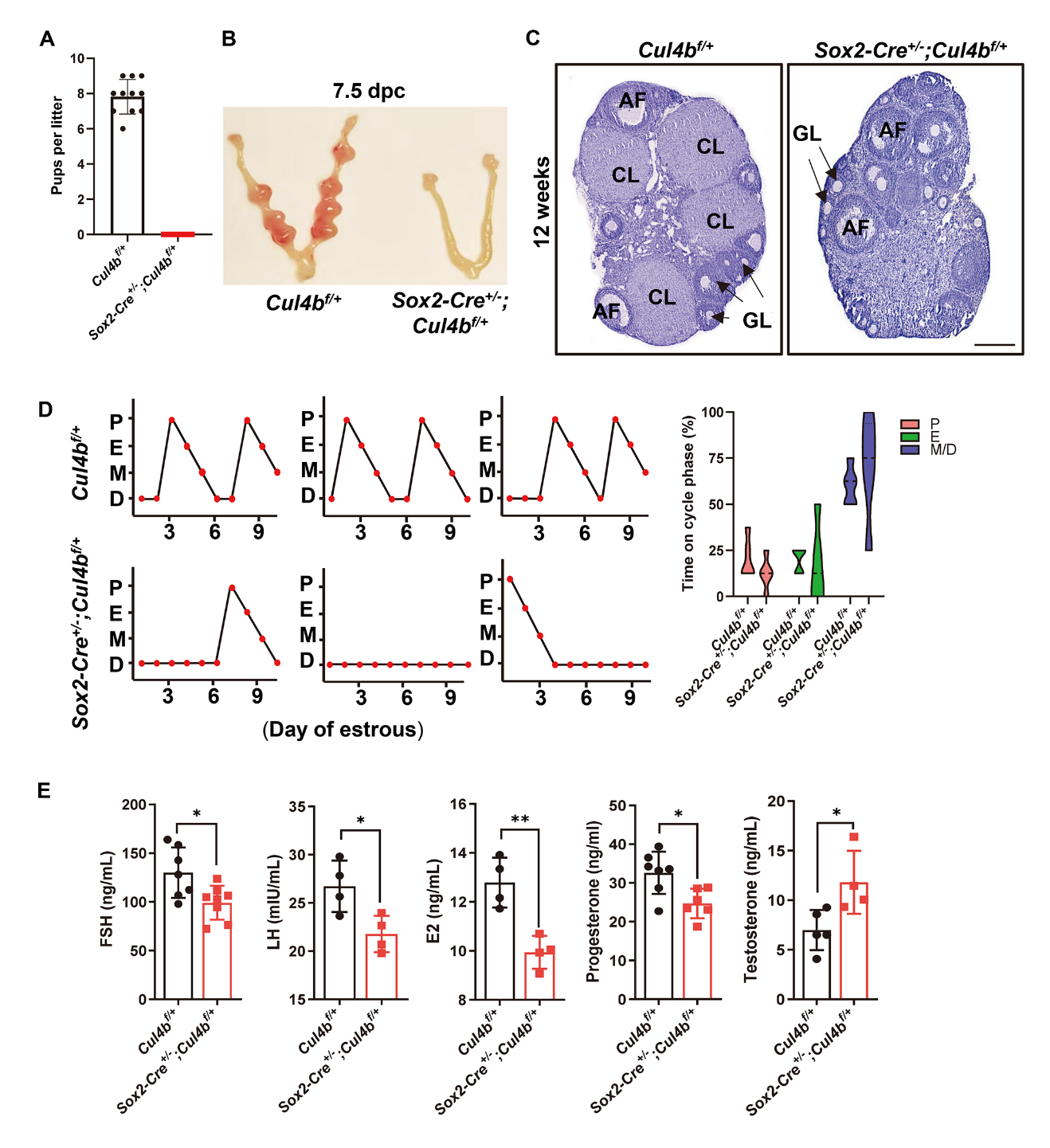


**Figure S1** Fetus in the uterus of *Cul4b^f/+^* and *Sox2-Cre^+/-^*;*Cul4b^f/+^* mice at 7.5 days post coitum (dpc).


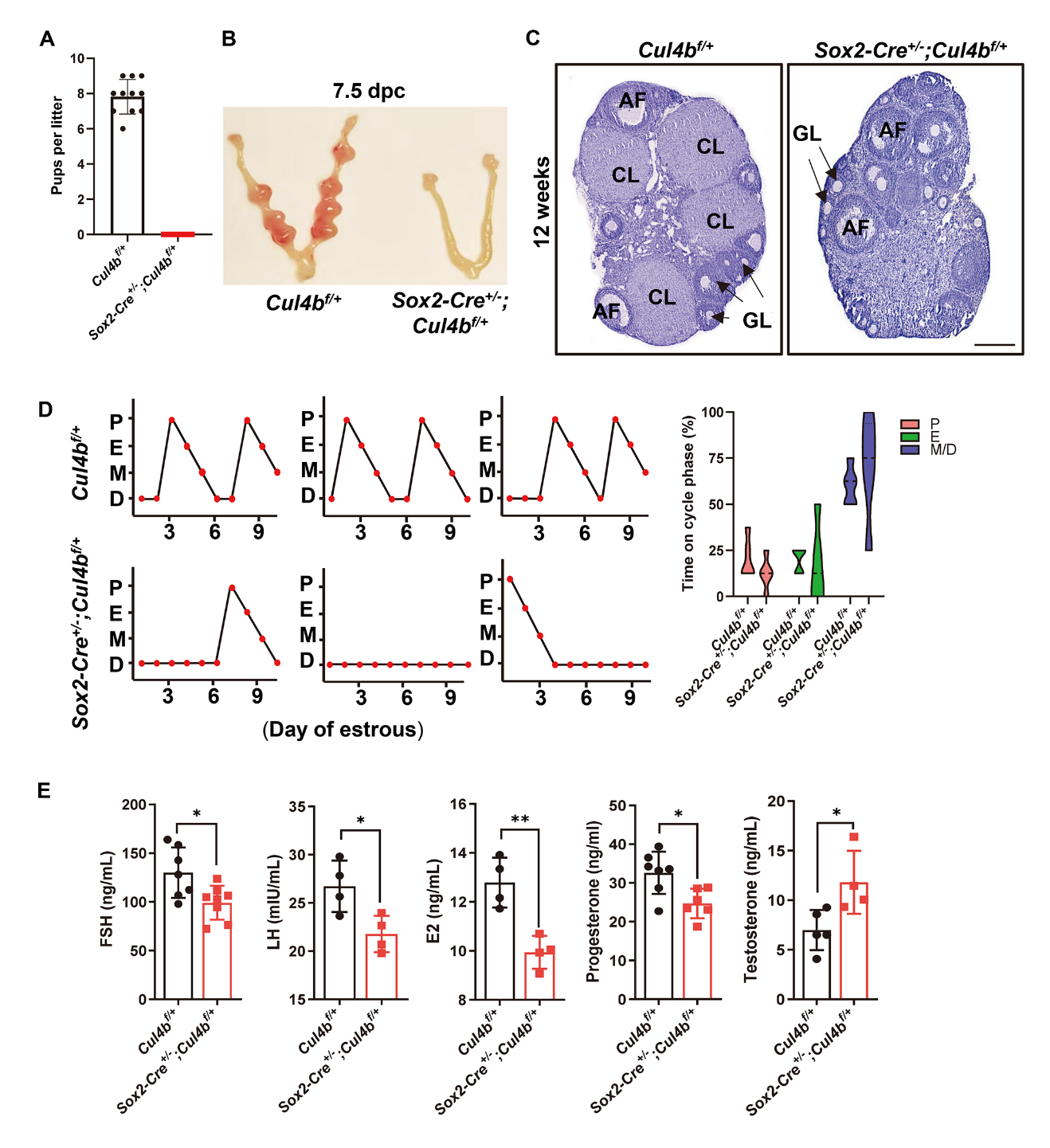


**Figure S2** Serum levels of FSH, LH, estradiol (E2), progesterone and testosterone in 8-week-old *Cul4b^f/+^* and *Sox2-Cre^+/-^*;*Cul4b^f/+^* mice. **p*<0.05; ***p*<0.01.


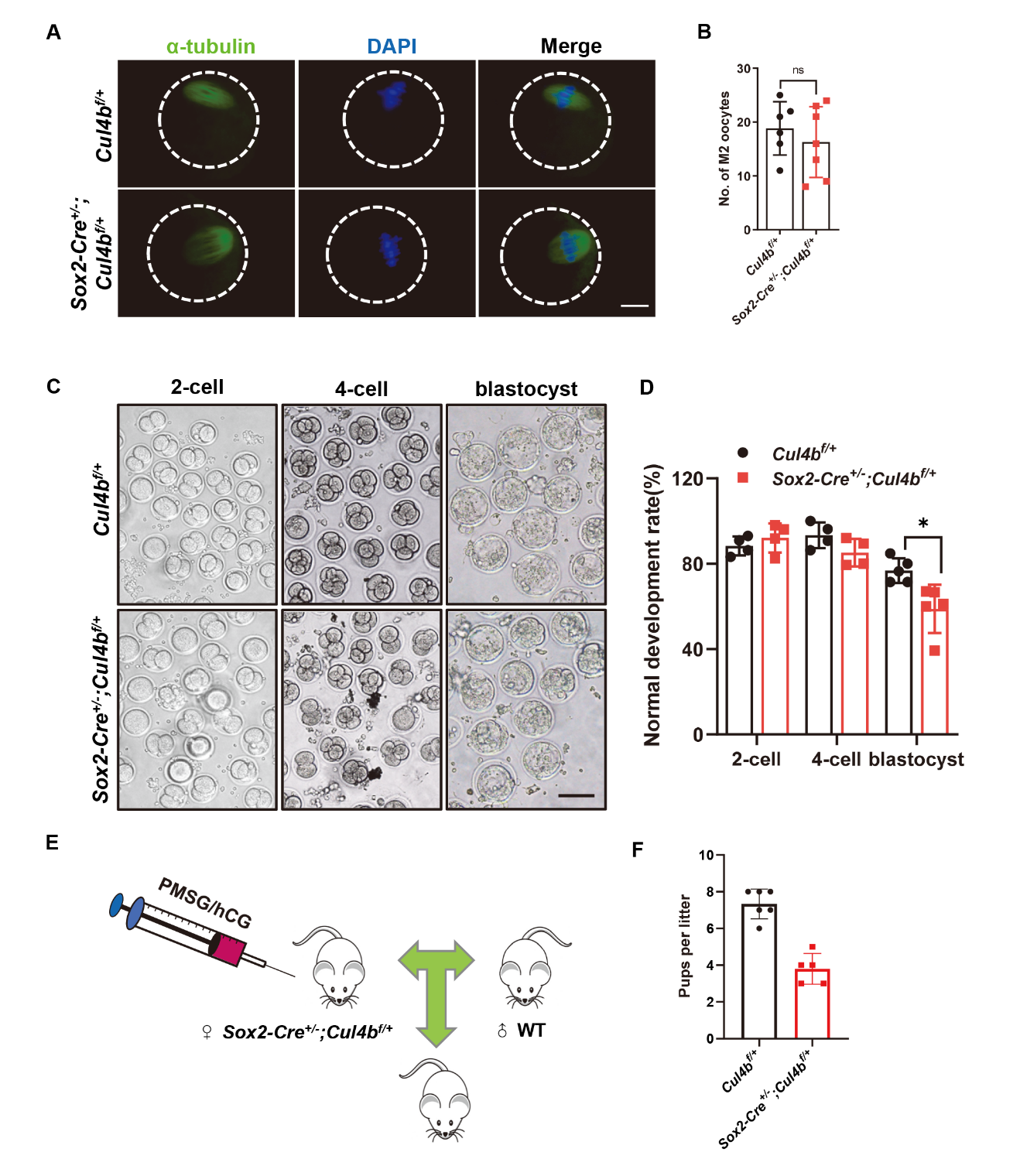


**Figure** **S3** Immunostaining of α-tubulin in meiotic oocytes of *Cul4b^f/+^* and *Sox2-Cre^+/-^*;*Cul4b^f/+^* mice. Scale bar, 25 μm.


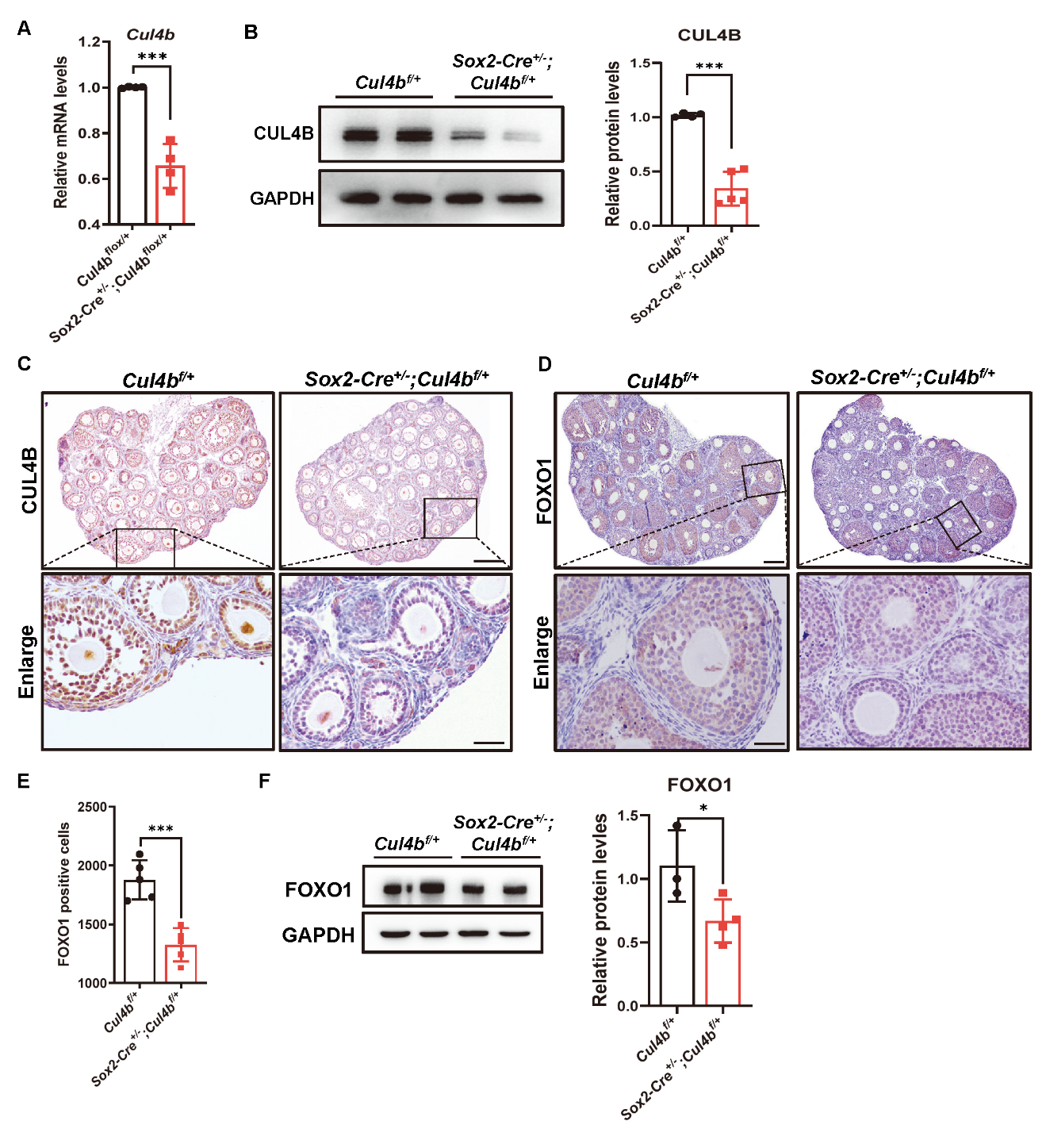


**Figure S4** The number of granulosa cells was decreased in the follicles of *Sox2-Cre^+/-^*;*Cul4b^f/+^* mice. (**A**) Relative *Cul4b* mRNA levels in the ovaries of *Cul4b^f/+^* and *Sox2-Cre^+/-^*;*Cul4b^f/+^* mice were determined by qPCR. (**B**) CUL4B protein levels in the ovaries of *Cul4b^f/+^* and *Sox2-Cre^+/-^*;*Cul4b^f/+^* mice were determined by Western blotting. (**C**) Representative CUL4B IHC images in the ovaries of *Cul4b^f/+^* and *Sox2-Cre^+/-^*;*Cul4b^f/+^* mice. Scale bars, 200 μm (upper) and 50 μm (lower). (**D**) Representative IHC images of FOXO1 in the ovaries of *Cul4b^f/+^* and *Sox2-Cre^+/-^*;*Cul4b^f/+^* mice. Scale bars, 200 μm (upper) and 50 μm (lower). (**E**) The numbers of FOXO1-positive granulosa cells in the follicles of *Cul4b^f/+^* and *Sox2-Cre^+/-^*;*Cul4b^f/+^* mice. (**F**) FOXO1 protein levels in the ovaries of *Cul4b^f/+^* and *Sox2-Cre^+/-^*;*Cul4b^f/+^* mice were determined by Western blotting. **p*<0.05; ****p*<0.001.


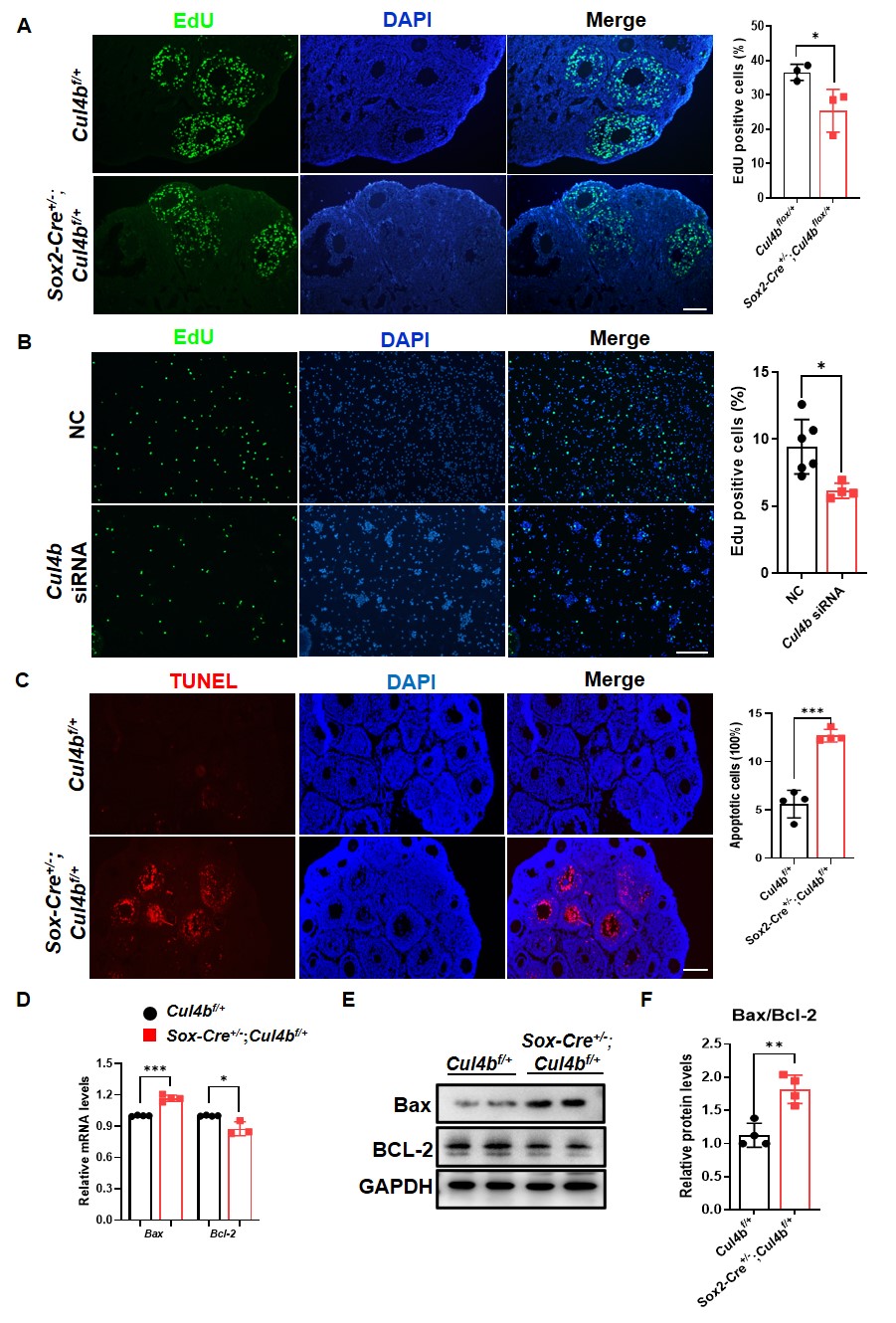
 **Figure S5** Decreased proliferation and increased apoptosis of granulosa cells in *Sox2-Cre^+/-^*;*Cul4b^f/+^* mice. (**A**) Cell proliferation was assayed by EdU staining in the ovaries of *Cul4b^f/+^* and *Sox2-Cre^+/-^*;*Cul4b^f/+^* mice, and the percentage of EdU-positive cells per follicle is shown. Scale bar, 100 μm. (**B**) Cell proliferation was assayed by EdU staining in NC and *Cul4b* knockdown primary granulosa cells, and the percentage of EdU-positive cells is shown. Scale bar, 200 μm. (**C**) Cell apoptosis was assayed by TUNEL assay in the ovaries of *Cul4b^f/+^* and *Sox2-Cre^+/-^*;*Cul4b^f/+^* mice, and the percentage of apoptotic cells per follicle is shown. Scale bar, 100 μm. (**D**) Relative mRNA levels of *Bax* and *Bcl-2* in the ovaries of *Cul4b^f/+^* and *Sox2-Cre^+/-^*;*Cul4b^f/+^* mice were determined by qPCR. (**E**-**F**) BAX and BCL-2 protein levels in the ovaries of *Cul4b^f/+^* and *Sox2-Cre^+/-^*;*Cul4b^f/+^* mice were determined by Western blotting. The relative expression of the BAX/BCL-2 protein is shown. **p*<0.05; ***p*<0.01; ****p*<0.001.


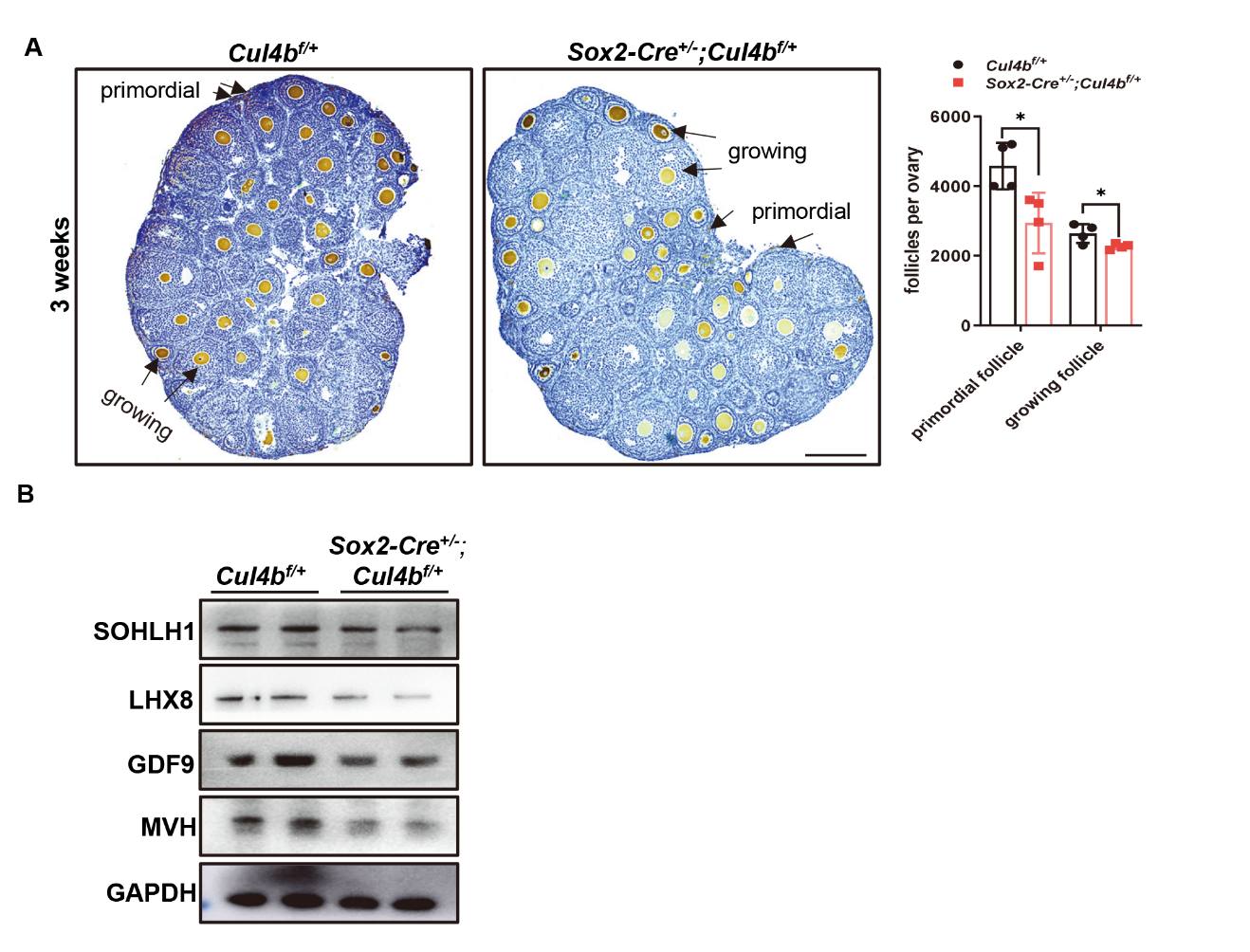


**Figure S6** Follicle development was impaired in the ovaries of *Sox2-Cre^+/-^*;*Cul4b^f/+^* mice. (**A**) Representative IHC images of MVH in the ovaries of *Cul4b^f/+^* and *Sox2-Cre^+/-^*;*Cul4b^f/+^* mice at 3 weeks. Scale bar, 100 μm. The numbers of primordial and growing follicles per ovary in *Cul4b^f/+^* and *Sox2-Cre^+/-^*;*Cul4b^f/+^* mice are shown. (**B**) SOHLH1, LHX8, GDF9 and MVH protein levels in the ovaries of *Cul4b^f/+^* and *Sox2-Cre^+/-^*;*Cul4b^f/+^* mice were determined by Western blotting. **p*<0.05.


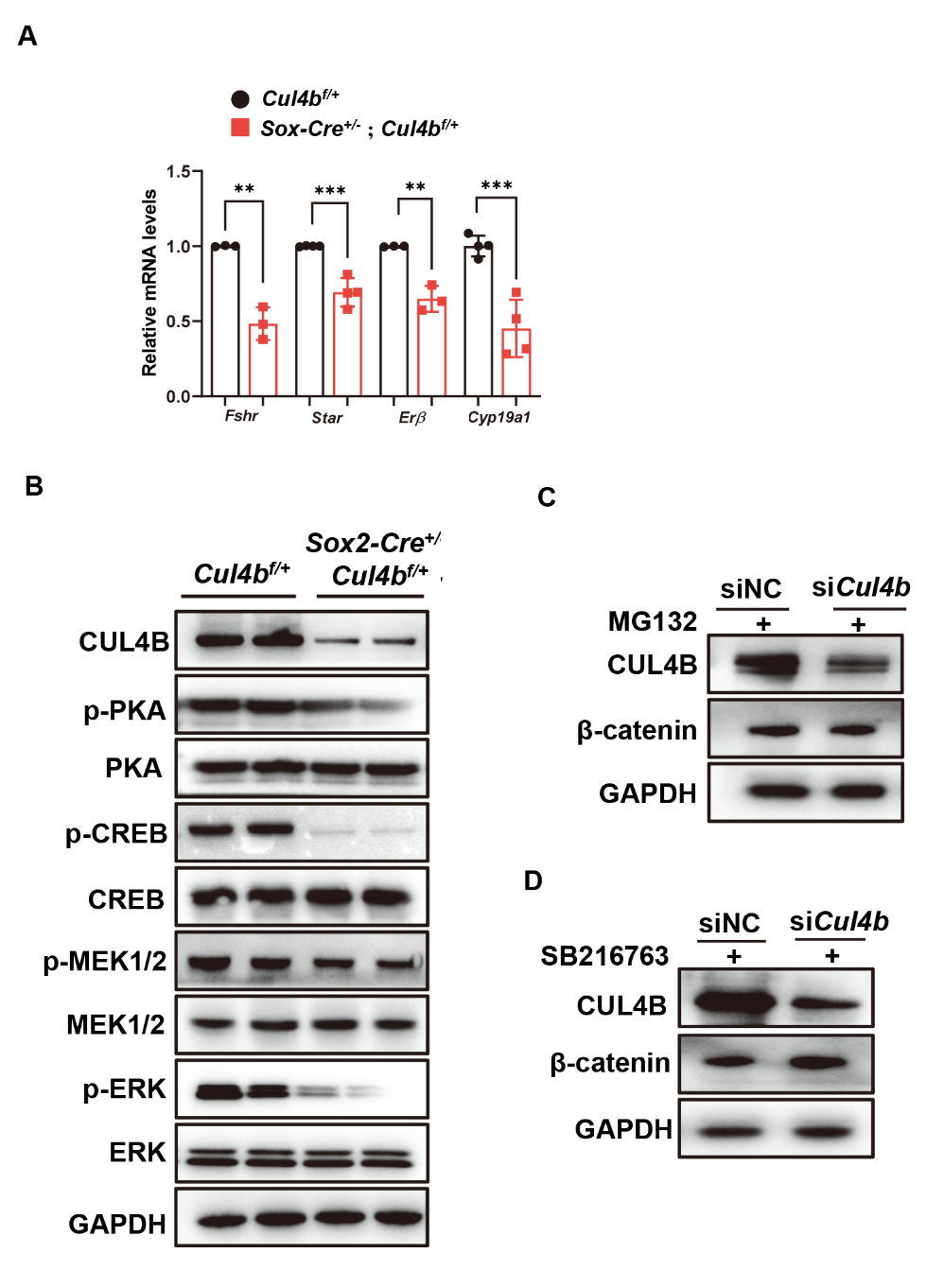


**Figure S7** CUL4B regulated the FSH/aromatase/estrogens loop. (**A**) Relative mRNA levels of *Fshr*, *Erbeta*, *Star* and *Cyp19a1* in the ovaries of *Cul4b^f/+^* and *Sox2-Cre^+/-^*;*Cul4b^f/+^* mice were determined by qPCR. (**B**) The phosphorylation of PKA, CREB, MEK and ERK in the ovaries of *Cul4b^f/+^* and *Sox2-Cre^+/-^*;*Cul4b^f/+^* mice was determined by Western blotting. (**C**) Control and *Cul4b*-knockdown KGN cells were cultured with MG132 for 2 hours, and cell lysates were analyzed for CUL4B and beta-catenin. (**D**) Control and *Cul4b*-knockdown KGN cells were cultured with SB216763 for 2 hours, and cell lysates were analyzed for CUL4B and beta-catenin. ***p*<0.01; ****p*<0.001.


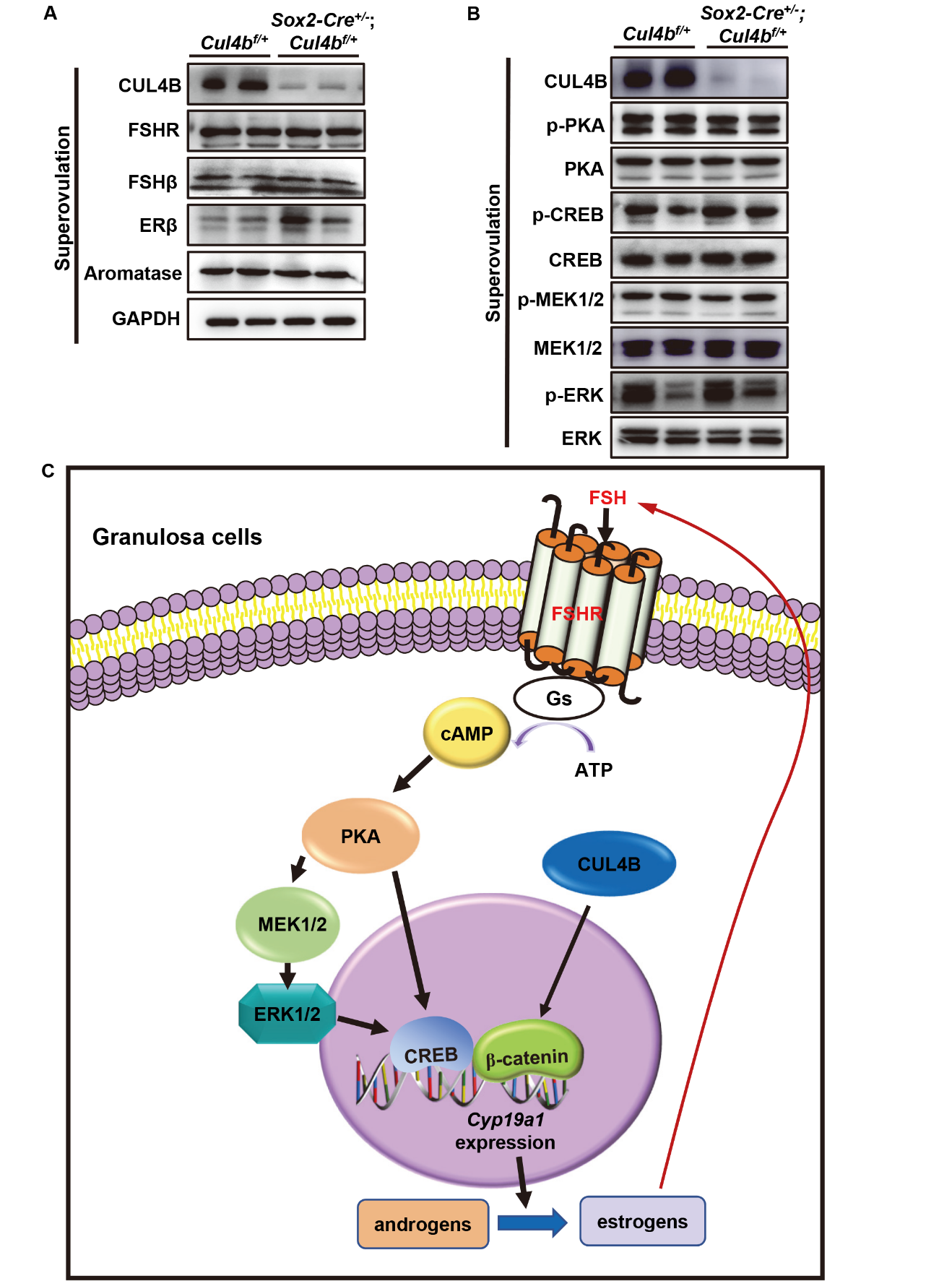


**Figure S8** The FSH/cAMP pathway were restored in the ovaries of superovulated *Sox2-Cre^+/-^*;*Cul4b^f/+^* mice. (**A**) The protein levels of FSHR, FSHβ, ERβ and aromatase in the ovaries of superovulated *Cul4b^f/+^* and *Sox2-Cre^+/-^*;*Cul4b^f/+^* mice were determined by Western blotting. (**B**) The phosphorylation of PKA, CREB, MEK and ERK in the ovaries of superovulated *Cul4b^f/+^* and *Sox2-Cre^+/-^*;*Cul4b^f/+^* mice was determined by Western blotting.

**Supplementary Tables**

**Supplementary Table 1.** Primers for quantitative real-time RT-PCR

| Primers | Sequences |
| --- | --- |
| *Cul4b* F | 5’-TATTAGTTGGCAAGAGTGCAT-3’ |
| *Cul4b* R | 5’-CCAGTAACCCATTGTCAGGAT-3’ |
| *Fshr* F | 5’-AAAAGCTTGTCGCCCTCATG-3’ |
| *Fshr* R | 5’-ACCATATCAGGACTCTGAGG-3’ |
| *Erꞵ* F | 5’-AGAGTCCCTGGTGTGAAGCAAG-3’ |
| *Erꞵ* R | 5’-GACAGCGCAGAAGTGAGCATC-3’ |
| *Star* F | 5’-CCGGGTGGATGGGTCAA-3’ |
| *Star* R | 5’-CACCTCTCCCTGCTGGATGTA-3’ |
| *Cyp19a1* F | 5’-AGCTGAGAAACTGGAAGACTG-3’ |
| *Cyp19a1* R | 5’-GAAGTACAGAGTGACCGACATG-3’ |

**Supplementary Table 2.** Antibodies

| Antibodies | Company | Dilution |
| --- | --- | --- |
| CUL4B | Sigma | 1:4000 |
| FOXO1 | CST | 1:1000 |
| FSHR | ZEN BIO | 1:500 |
| FSHꞵ | BOSTER | 1:500 |
| Aromatase | Abcam | 1:1000 |
| GAPDH | Proteintech | 1:2000 |
| MVH | Abcam | 1:1000 |
| SOHLH1 | Abcam | 1:1000 |
| LHX8 | Abcam | 1:1000 |
| GDF9 | Abcam | 1:500 |
| p-PKA | CST | 1:1000 |
| PKA | Abcam | 1:1000 |
| p-CREB | CST | 1:1000 |
| CREB | CST | 1:1000 |
| p-MEK1/2 | CST | 1:1000 |
| MEK1/2 | Proteintech | 1:1000 |
| p-ERK | CST | 1:1000 |
| ERK | Abcam | 1:1000 |
| ꞵ-catenin | CST | 1:1000 |
| p-GSK3ꞵ | CST | 1:1000 |
| GSK3ꞵ | Abcam | 1:1000 |
